# Modification of Polydiallyldimethylammonium Chloride with Sodium Polystyrenesulfonate Dramatically Changes the Resistance of Polymer-Based Coatings towards Wash-Off from Both Hydrophilic and Hydrophobic Surfaces

**DOI:** 10.3390/polym14061247

**Published:** 2022-03-19

**Authors:** Vladislava A. Pigareva, Ivan N. Senchikhin, Anastasia V. Bolshakova, Andrey V. Sybachin

**Affiliations:** 1 Chemistry Department, Lomonosov Moscow State University, 119991 Moscow, Russia; vla_dislava@mail.ru (V.A.P.); bolshakova@belozersky.msu.ru (A.V.B.); 2 Frumkin Institute of Physical Chemistry and Electrochemistry, Russian Academy of Sciences, 119071 Moscow, Russia; isenchikhin@gmail.com

**Keywords:** polycation, polyanion, interpolyelectrolyte complex, coating, sodium polystyrenesulfonate, polydiallyldimethylammonium chloride, AFM, SEM

## Abstract

Polymer coatings based on polycations represent a perspective class of protective antimicrobial coatings. Polydiallyldimethylammonium chloride (PDADMAC) and its water-soluble complexes with sodium polystyrenesulfonate (PSS) were studied by means of dynamic light-scattering, laser microelectrophoresis and turbidimetry. It was shown that addition of six mol.% of polyanion to polycation results in formation of interpolyelectrolyte complex (IPEC) that was stable towards phase separation in water-salt media with a concentration of salts (NaCl, CaCl_2_, Na_2_SO_4_, MgSO_4_) up to 0.5 M. Most of the polyelectrolyte coatings are made by layer-by-layer deposition. The utilization of water-soluble IPEC for the direct deposition on the surface was studied. The coatings from the PDADMAC and the PSS/PDADMAC complex were formed on the surfaces of hydrophilic glass and hydrophobic polyvinylchloride. It was found that formation IPEC allows one to increase the stability of the coating towards wash-off with water in comparison to individual PDADMAC coating on both types of substrates. The visualization of the coatings was performed by atomic force microscopy and scanning electron microscopy.

## 1. Introduction

Polymers are widely used as functional coatings for regulating adhesive properties, imparting anticorrosive properties, electrical insulation, etc., [1,2,3,4]. Among the areas of application of polymer coatings, it is especially worth highlighting the effect of coatings with antibacterial properties [5,6]. In such systems, polymers perform various functions. Antifouling coatings prevent the adsorption of bacteria to the treated surface [7]. The polymers may be part of the composition for making coatings that can prevent the spread of adsorbed bacteria and prevent the formation of a biofilm [8]. Furthermore, polymer coatings are capable of destroying adsorbed bacteria, i.e., they exhibit biocidal properties [9,10]. In this case, macromolecules can serve as a matrix, in which a low molecular weight biocide or biocidal nanoparticles are distributed, they can also contain covalently attached biocidal molecules, and finally they may independently exert a biocidal effect due to their functional groups [11,12,13,14,15]. Among the polymers with biocidal properties, one should single out the class of polycations—synthetic or natural polyelectrolytes that carry a positive charge in the monomer unit [16]. One such representative of polycations is polydiallyldimethylammonium chloride (PDADMAC), which was shown to possess antimicrobial activity [17]. One of the advantages of PDADMAC is that it has good solubility in water, as well as in the presence of a pH-independent quaternized amino group. This allows one to use various methods of forming coatings from solutions by dip-coating, solution deposition on the surface with further drying, spraying, and so on [18,19,20]. On the other hand, good solubility in water can lead to effective rinsing of coatings upon contact of the treated surface with water. An important aspect for the formation of a strong polycation-surface contact is the presence of negatively charged functional groups on the treated object. In this case, a strong electrostatic complex could be formed. On glass surfaces, such an interaction is carried out mainly due to silanol groups [21]. Another aspect that affects the formation of a coating from aqueous solutions is the hydrophilicity/hydrophilicity of the treated substrate [22]. It is obvious that lipophilic surfaces prevent the distribution of an aqueous solution over their area. In order to ensure good contact of the polycation with such a surface, it is necessary to partially hydrophobize it. To impart hydrophobicity to a polycation, it is possible to synthesize a macromolecule by including lipophilic blocks (for example, polymeric soaps) in it [23,24]. However, a simpler and more efficient method of partial hydrophobization of polyactions is the formation of their complexes with oppositely charged polyanions. The product of such an interaction, the interpolyelectrolyte complex (IPEC), is an amphiphilic compound containing hydrophilic regions due to free charged groups and hydrophobic regions of a compensated charge [25,26]. The solubility of IPEC in water depends on many factors—the ratio of charged groups of polymers, their chemical nature, the ionic strength of the solution, the pH of the medium, etc., [27,28,29,30,31,32,33]. Important parameters are the degree of polymerization of the components of the IPEC of polyelectrolytes, as well as their ratio [34]. In comparison to layer-by-layer deposition of the polyelectrolytes on the substrate, with the aim of obtaining IPEC coating, in this work we discuss the possibility of using water-soluble IPEC for the direct deposition on the surface. Attention is paid to the formation of water-soluble complexes based on pH-independent PDADMAC and sodium polystyrene sulfonate (PSS), the stability of the complexes in aqueous-salt media, and for the optimal composition, the structure and properties of coatings formed from solution, formed both on hydrophilic and on hydrophobic surfaces are investigated.

## 2. Materials and Methods

### 2.1. Materials

Polydiallyldimethylammonium chloride (PDADMAC) with average molecular mass Mw = 400–500 kDa and sodium polystyrene sulfonate (PSS) with average molecular mass Mw = 70 kDa were used as received from Sigma-Aldrich (St. Louis, MO, USA). Structures of polymers could be found in Supplementary Materials (see Figure S1). Sodium chloride (NaCl), sodium sulfate (Na_2_SO_4_), calcium chloride (CaCl_2_), magnesium sulfate (MgSO_4_), tris(hydroxymethyl)aminomethane (Tris) with analytical grade from Reachem (Moscow, Russia) were used as received. Glass cover slips with an area of 2.25 cm^2^ were used in the experiments to wash-off the polymer films. Cleaning and preparation of the surface was carried out as follows: the coverslip was dipped in methanol and vigorously shaken for a minute. After that, the glass was treated with 1 M KOH solution, then washed with bidistilled water and dried.

Optical borosilicate glasses with a diameter of 15 mm from Edmund Optics (Barrington, NJ, USA) were used as substrate for the microscopy experiments.

Polyvinylchloride (PVC) samples were prepared from food processing conveyor belts with a polyvinylchloride working surface 1 N 71+ series by Nitta (Osaka, Japan).

The substrates were cleaned with ethanol and then washed with bidistilled water prior to use.

Bidistilled water with conductivity 0.5 μS/cm was used in all the experiments.

### 2.2. IPEC Preparation

The formation of IPEC was performed by the titration of the PDADMAC solution in Tris buffer with pH 7.0 and concentration of NaCl 0.005 M with PSS solution under stirring conditions to provide homogenous distribution of the macromolecules. The homogeneity of the solution was controlled by turbidimetry.

### 2.3. PDADMAC and IPEC Coatings Wash-Off Procedure

Freshly cleaned substrate (glass coverslip or PVC sample) with 2.25 cm^2^ area was weighed. The 200 μL aliquote of the 20 mg/mL solution of polymer or IPEC was deposited on the substrate so that all the area was covered with the solution. The sample was left to dry overnight. The prepared sample was weighted once again and the mass of the film was calculated as the difference between the masses of substrate with film and bare substrate. Each cycle of wash-off was as follows: 200 μL of water was applied to the glass, with coating, so that it completely covered the surface of the film. After one minute of incubation the liquid was eliminated and the sample was left to dry. The sample was weighted and the mass loss was calculated.

### 2.4. Preparation of IPEC Coatings for the Microscopy Analysis

The sample surfaces were prepared as described in the previous section. The cleaned substrate (glass or PVC) was immersed in a 20 mg/mL IPEC solution for two minutes. The IPEC-coated substrate was then thoroughly washed with water in a beaker. Then the samples were left to dry on the air. The resulted coatings were analyzed by a scanning electron microscopy (SEM) and an atomic force microscope (AFM).

### 2.5. Methods

Turbidimetry experiments were carried with the use of spectrophotometer UV-mini 1240 Hitachi (Tokyo, Japan) at wavelength λ = 400 nm. The acrylic cuvettes with 10 mm path length were used.

The diffusion coefficients for the PDADMAC and its complexes were obtained by dynamic light-scattering measurements were carried using a Complex laser light goniometer by Photocor Instruments (Moscow, Russia) equipped with a He−Ne laser and data processing was performed using DynaLS software version 2.7.1 [35]. Diffusion coefficients were recalculated into mean hydrodynamic diameters with the Stokes−Einstein equation.

Electrophoretic mobility (EPM) of PDADMAC and its complexes were determined in a thermostatic cell by laser microelectrophoresis using Brookhaven ZetaPlus equipment with the software supplied by the Brookhaven (Brookhaven, GA, USA). 

The gravimetry analysis was made using precise balances VLA-120 M by Gosmetr (Saint Petersburg, Russia).

The morphology of IPEC coatings was determined by SEM using Quanta 650 FEG by FEI (Hillsboro, OR, USA) equipped with a field-emission cathode. Samples were fixed on an aluminum holder using double-sided conductive carbon tape. Then the samples were placed in an instrument chamber and were investigated under a high vacuum at an accelerating voltage of 2 kV. SEM image processing was performed using FemtoScan Online software version 4.8 (http://www.femtoscanonline.nanoscopy.ru, (accessed on 14 February 2022)).

The structure of the IPEC films on the glass and PVC surfaces was made with AFMMultimode Nanoscope V by Veeco (Plainview, NY, USA) working in tapping mode. We used polysilicon cantilevers with high accuracy composite probes HA-FM by TipsNano, (Tallinn, Estonia) with resonance frequency 76 KHz, and with a Q-factor of about 280.

Statistical analysis. The average results of at least five experiments are presented as mean values.

## 3. Results

### 3.1. Formation of Water-Soluble IPEC

**Figure 1:**
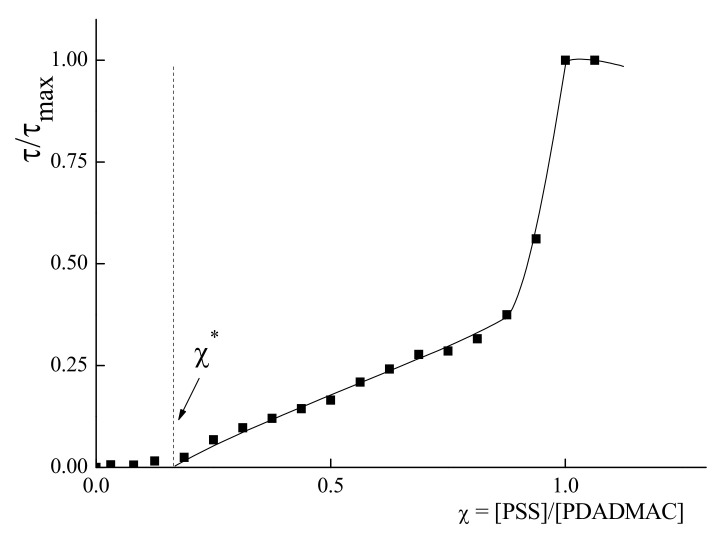
Turbidimetric titration curve for the PDADMAC mixture with PSS. C_NaCl_ = 0.005 M, C_PDADMAC_ = 4 × 10^−4^ base-mol/L, pH 7.0.

The results of the turbidimetric titration of PDADMAC solution with PSS are presented in Figure 1 as dependence of relative turbidity (τ/τ_max_) upon the ratio of the anionic and cationic groups of the polymers χ = [PSS]/[PDADMAC]. No change in turbidity was observed up to critical composition χ* = 0.16 corresponding to formation of water-soluble IPEC. Further addition of the PSS to the PDADMAC results in an increase in the turbidity, indicating the formation of a heterogeneous system, i.g. phase separation. After reaching χ = 0.8, the sharp rise of the turbidity was detected. Maximal value of the turbidity was reached at χ = 1, reflecting the formation of a stoichiometric insoluble complex. So, below we will consider only complexes with χ ≤ 0.16 as completely water-soluble. 

### 3.2. Phase Sepatation in IPEC Water-Salt Solutions

**Figure 2:**
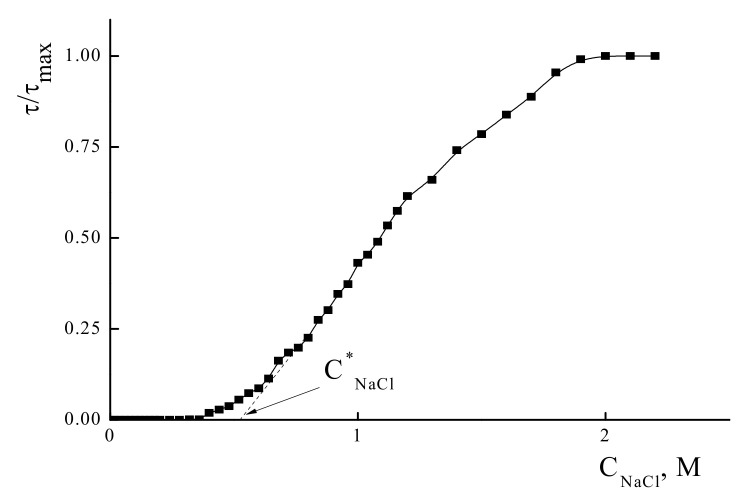
Turbidimetric titration curve for the PDADMAC/PSS IPEC mixture with NaCl. C_PDADMAC_ = 4 × 10^−4^ base-mol/L, C_PSS_ = 2.4 × 10^−5^ base-mol/L pH 7.0.

Water-soluble IPECs are quite sensitive to the ionic strength of the solution. An increase in the last could result in salt-induced phase separation of the IPEC [34,35,36]. First, the role of the monovalent salt- NaCl, concentration was studied. Figure 2 represents typical curve of the titration of water-soluble IPEC with salt. The addition of NaCl to the solution of the PSS/PDADMAC IPEC with χ = 0.6 did not result in the change of turbidity up to a critical salt concentration C*_NaCl_ = 0.5 M. Further increase in salt concentration results in the formation of a heterogeneous system. No decrease in the turbidity that could reflect dissociation of IPEC to the individual polyelectrolytes was observed, even at C_NaCl_ = 2 M.

**Figure 3:**
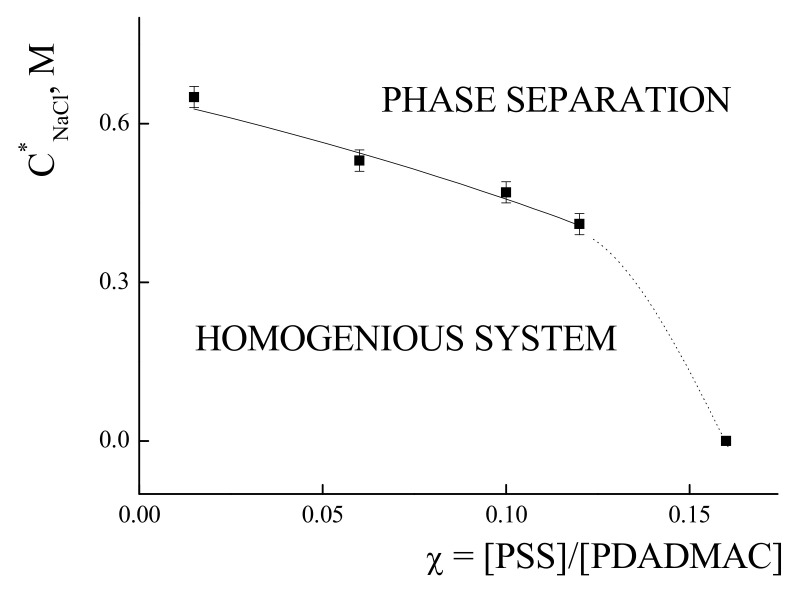
Values of critical salt concentration of NaCl corresponding to the onset of phase separations in IPECs solutions prepared by mixing PSS with solutions of PDADMAC versus IPEC composition χ. C_PDADMAC_ = 4 × 10^−4^ base-mol/L, pH 7.0.

The values of the C*_NaCl_ were obtained for the IPECs with χ values from 0.015 to 0.16 (see details in Supplementary Materials Figure S2). The results are presented in Figure 3. The decrease in the C*_NaCl_ from 0.62 to 0.45 was observed with changing χ from 0.015 to 0.12, with a further sharp decrease to C*_NaCl_ = 0.005 M for the IPEC with χ*.

**Figure 4:**
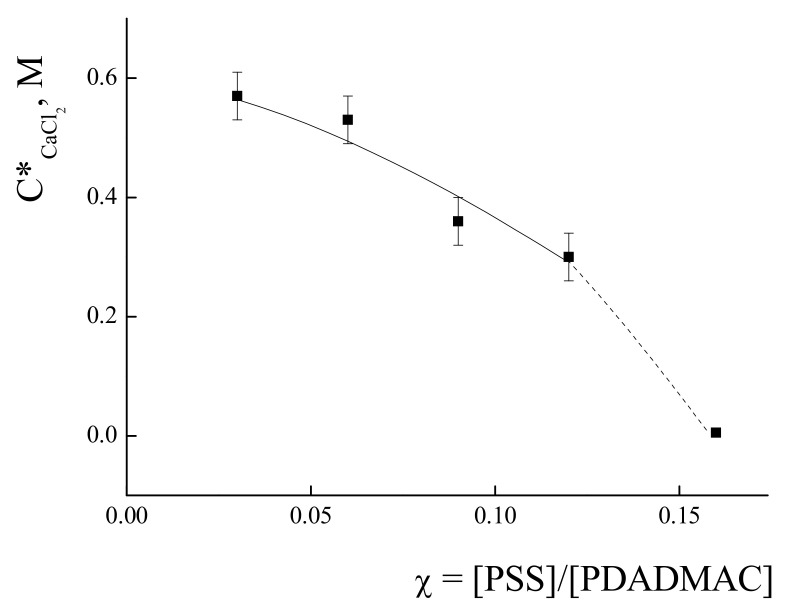
Values of critical salt concentration of CaCl_2_ corresponding to the onset of phase separations in IPECs solutions prepared by mixing PSS with solutions of PDADMAC versus IPEC composition χ. C_PDADMAC_ = 4 × 10^−4^ base-mol/L, pH 7.0.

The phase separation was studied for the salts with bivalent ions—CaCl_2_ (see details in Supplementary Materials Figure S3), Na_2_SO_4_ and MgSO_4_. The dependence of critical salt concentration C*_CaCl2_ upon IPEC composition is presented in Figure 4. The progressive decrease in the C*_CaCl2_ values from 0.57 M to 0.3 M was observed for the IPECs with an increase in χ from 0.03 to 0.12, respectively. A further increase in χ results in a sharp decrease in C*_CaCl2_ to the value of zero. With the use of Na_2_SO_4_ and MgSO_4_ as the parameters controlling the ionic strength of the IPECs solutions, no phase separation was observed for up to 1 M of concentration of salts for all complexes with χ from 0.03 to 0.12. 

According to the data presented in Figure 3 and Figure 4, the IPEC with χ = 0.06 was chosen for further investigation as it is complex with relatively high content of PSS and high stability towards phase separations in water-salt solutions. The critical values of the salt concentrations for this IPEC were found to be C*_NaCl_ = 0.5 M and C*_CaCl2_ = 0.5 M.

### 3.3. Characterization of the IPEC with χ = 0.06 in Water-Salt Media

**Figure 5:**
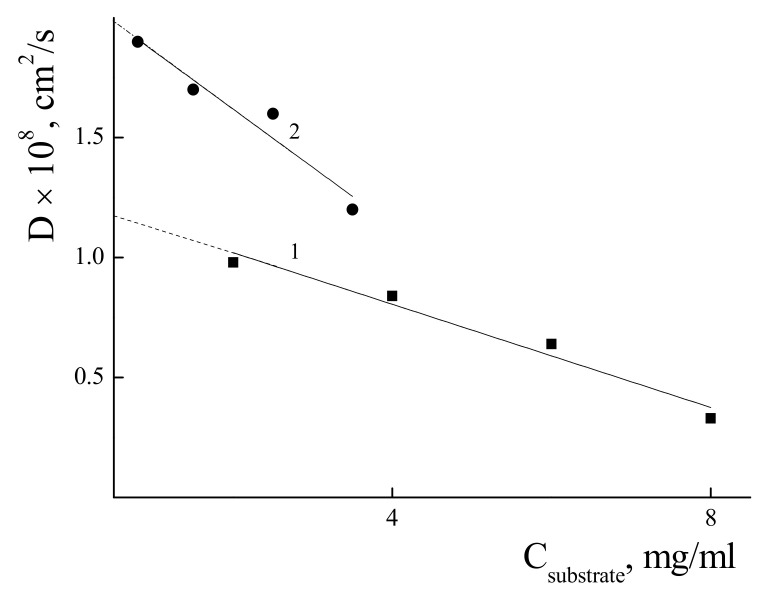
Dependence of the diffusion coefficient upon the concentration of a solution of PDADMAC (1) and IPEC (2) in a water-salt media. pH 7; C_NaCl_ = 0.05 M.

The sizes of the PDADMAC and IPEC with χ = 0.06 were studied by means of DLS. The dependences of the diffusion coefficients (D) of polycation and its complex upon their concentration were measured in 0.05 M of NaCl solution to avoid a polyelectrolyte swelling effect. The results are presented in Figure 5. To determine the hydrodynamic radii, diffusion coefficients were measured for a series of solutions with different concentration. Extrapolation of the concentration dependence of D to zero concentration allowed us to estimate the resulting D_0_ value, which was used in calculations using the Stokes−Einstein equation (see the linearization parameters in Supplementary Materials Figure S4). The calculated values of the hydrodynamic radii were 180 nm and 110 nm for the PDADMAC and IPEC with χ = 0.06, respectively. Hydrophobization of the PDADMAC with PSS results in compactization of the macromolecure.

The values of EPM reflecting the density of the surface charge of the species were measured to be 3.4 ± 0.2 (µm/s)/(V/cm) for the both samples—PDADMAC and IPEC. 

### 3.4. Stucture and Properties of the IPEC Coatings

#### 3.4.1. The Resistance of the Polymer Coatings towards Wash-Off with Water

**Figure 6:**
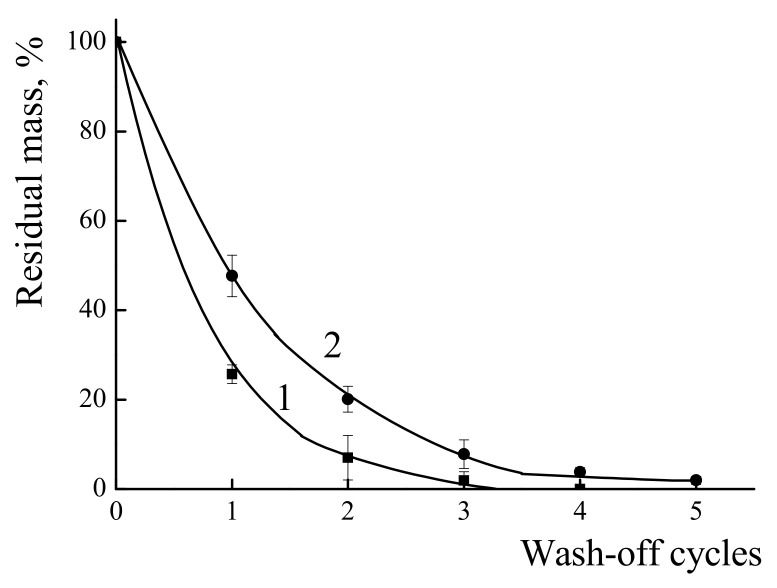
Dependence of the percentage of the retained mass of films formed from PDADMAC (1) and IPEC (2) on a glass surface on the number of wash-off cycles.

The resistance of the polymers films towards wash-off with water was monitored by the weight loss of the sample. The results are presented in Figure 6. For the individual PDADMAC, about 75% of weight loss was observed after the first wash-off cycle and almost all polycation had vanished after four cycles of the wash-off. In contrast, the IPEC with χ = 0.06 retained about 50% of its film mass after the first wash-off and 4% after four cycles. Even after five cycles the remaining IPEC could be detected on the glass surface.

**Figure 7:**
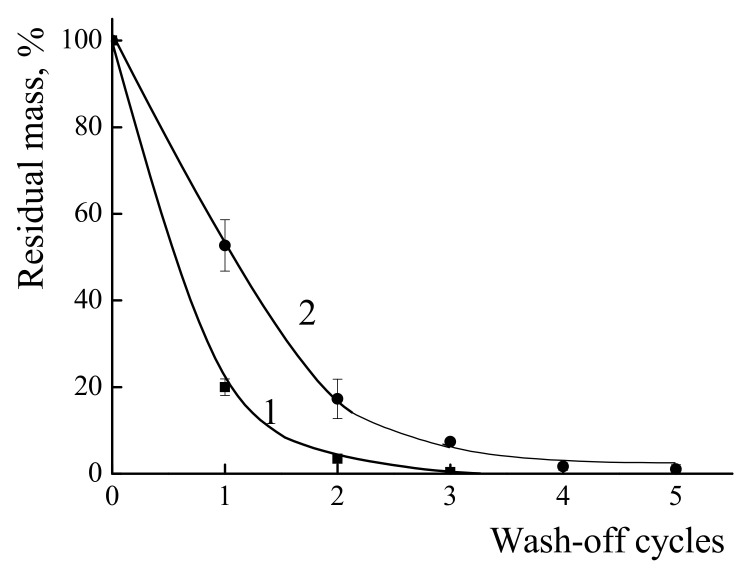
Dependence of the percentage of the preserved mass of films formed from PDADMAC (1) and IPEC (2) on a PVC surface on the number of wash-off cycles.

The same procedure was applied to study the behavior of the PDADMAC and IPEC with χ = 0.06 coatings on the PVC surface. The results of the wash-off investigation are presented in Figure 7. For the individual PDADMAC, about 80% of weight loss was observed after the first wash-off cycle and almost all polycation had vanished after three cycles of the wash-off. In contrast the IPEC with χ = 0.06 retained about 50% of its film mass after the first wash-off and 7.5% after three cycles of wash-off. Even after five cycles the remaining IPEC could be detected on the PVC surface.

#### 3.4.2. The Structure of the IPEC Coatings

**Figure 8:**
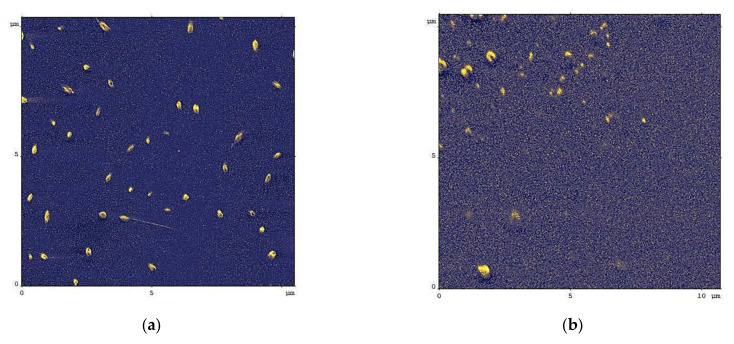
SEM images of coatings from IPEC with χ = 0.06 on the substrate: (**a**) glass; (**b**) PVC.

The SEM images of the IPEC films are presented in Figure 8. On both surfaces, IPEC films represent smooth continuous coatings with the IPEC particles or IPEC agglomerates adsorbed on them. The similar images were obtained even using 0.01 mg/mL solutions of IPEC (the data not shown).

The detailed insight in structure of the IPEC films on the glass and PVC surfaces was made with AFM. The IPEC-coated substrate was then thoroughly washed with water in a beaker and dried in air. In general, IPEC formed smooth films on both glass and PVC surfaces. So, the areas with defects were searched for the analysis of the thickness of the IPEC layers.

**Figure 9:**
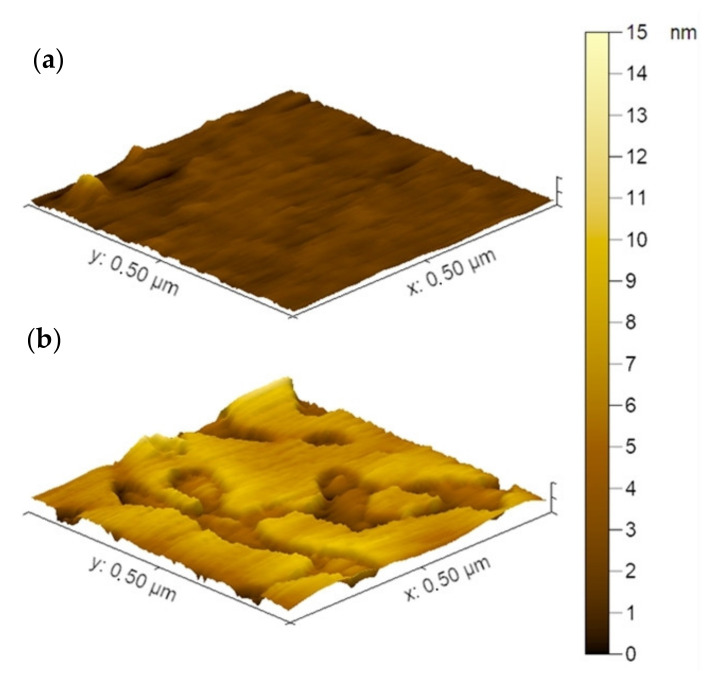
AFM images of coatings from IPEC with χ = 0.06 on the glass (**a**) and PVC (**b**) substrate.

In Figure 9a the typical AFM image of the coatings from IPEC with χ = 0.06 on the glass substrate is presented. The analysis of the defects was made using Gwyddion software version 2.60: (see the normalized distribution of the sizes in Supplementary Materials Figure S5). The average depth of the defects in the IPEC layer on the glass was found to be of 3.8 nm, which can be attributed to the thickness of the IPEC layer on the glass. 

In Figure 9b the typical AFM image of the coatings from IPEC with χ = 0.06 on the PVC substrate is presented. The relief of the surface, in Figure 9, have species with an average height of 6 nm that could be attributed to the layer of the IPEC on the PVC. 

## 4. Discussion

The water-soluble IPECs of PDADMAC as a host polyelectrolyte and PSS as a guest polyelectrolyte may be obtained in a very narrow interval of PSS to PDADMAC molar ratioχ. In comparison to previously reported data on the complexation of PSS with PDADMAC, one could expect the formation of water-soluble complexes in the range of χ up to 0.8 [37,38]. So, the obtained value χ* = 0.16 seems to be very low at first sight. However, the slope of the section of the turbidimetric dependence on Figure 1 in the region of χ values from 0.16 to 0.8 seems to be conditioned by the broad polydispersity of the macromolecules, as a fraction with relatively low molecular weight cannot ensure sufficient solubility for the complex. This means that for the χ values, from 0.16 to 0.8, there could be general fraction of water-soluble IPEC. These results are in good agreement with the fact demonstrated by Dautzenberg, that at low ionic strength the formation complexes between PDADMAC and short chain PSS is more favorable than with long chain PSS [39]. In addition the resulted aggregates are more governed by kinetics than by thermodynamics. Nevertheless, only complexes with χ ≤ 0.16 in the system under investigation could be considered as completely water-soluble. 

Water-soluble IPEC are affected by the presence of simple salts in solution. The IPEC structure should be considered as the dynamic system with areas of dense salt bonds between oppositive charged polyelectrolytes and areas with local excess to both cationic and anionic units [40,41,42]. So, the impact of the simple salts on the IPEC should be discussed from two general points of view. First, an increase in the ionic strength of the solution affects the counterion condensation in areas of non-compensated chains fragments [43]. Second, the addition of salt results in a weakening of the interpolyelectrolyte bonds, due to competitive reactions between macromolecules and counterions [40,44]. As a result, an increase in the ionic strength in IPEC solution could induce phase separation. It should be stressed that different ions may have specific affinity to the charged groups of the polyelectrolytes [45,46]. So, the influence of monovalent and bivalent ions’ concentration on the possibility to form water-soluble PSS/PDADMAC complexes was studied. It should be pointed out that the chloride ion was found to have a determinant value on the phase separation in solutions of the studied IPECs. The critical salt concentrations of NaCl and CaCl_2_, for the IPECs with the same χ value, did not differ reasonably. Substitution of a Cl^−^ ion to an SO_4_^2−^ ion resulted in significantly expanded diapason of the existence of water-soluble IPECs. It seems that phase separation is more determined by the interaction of small ions with a host polyelectrolyte. Moreover, for the PSS/PDADMAC complexes, the values of the critical salt concentrations were found to be higher than for those previously reported for the complexes of quaternized polyamines and polyanions with carboxyl groups [34]. This may be connected with the formation of relatively strong interpolyelectrolyte bonds for the quaternized aminogroups groups and sulfonate groups in comparison to carboxyl groups. These findings allow one to choose the composition of the PSS/PDADMAC based IPEC that will have the desired hydrophilic/hydrophobic balance, and will be stable towards phase separation in water-salt media.

Deposition of the water solution of PDADMAC or IPEC with χ = 0.06 on the surface of hydrophilic glass or hydrophobic PVC results in the formation of a polymer coating. However, a coating formed by the individual polycation could be easily removed by wash-off with water. It takes more cycles of wash-off to almost completely remove PDADMAC from hydrophilic surface than from a hydrophobic one. However, the modification of PDADMAC with only six mol.% of PSS results in formation of the coating with good resistance to wash-off on both types of surfaces (glass and PVC). Surface charge density of the IPEC with χ = 0.06 is equal to the surface charge density of PDADMAC, hence conditioning effective adsorption on hydrophilic glass with an anionic charged surface. On the other hand, a hydrophobic area of the IPEC provides effective adsorption on the hydrophobic PVC surface. In addition, hydrophobic interactions in the coating results in the formation of a layer resistant towards wash-off with water. The forming coatings represent by itself a continuous film with on average four and six nm thickness on the glass and PVC substrate, respectively. These results are in good agreement with the data obtained for the PSS/PDADMAC bilayers obtained by the layer-by-layer technique of D. Kovačević et al., and the results of simulation for the multilayers from oligo-PSS and oligo PDADMAC by Sanchez et al. [46,47].

## 5. Conclusions

Modification of the cationic PDADMAC macromolecule with oppositive charged PSS, so that only a 0.06 fraction of the charged aminogroups was compensated, resulted in the formation of a water-soluble IPEC with almost the following characteristics. The hydrodynamic radius of the complex was 110 nm, while for the PDADMAC, this value corresponded to 180 nm. At the same time, the surface charge density for the IPEC was the same as for the individual polycation—the EPM value corresponded to 3.4 (µm/s)/(V/cm). Thus, this partial neutralization of PDADMAC did not reasonably effect the charge of the resulted IPEC. The obtained IPEC has shown excellent resistance towards dissociation and phase separation in water-salt media formed by various salts. No phase separation occurs in IPEC with χ = 0.06 up to 0.5 M of either NaCl or CaCl_2_. Moreover, IPEC with χ = 0.06 retained homogeneity, even in the concentrated solution of Na_2_SO_4_ and MgSO_4_. This IPEC, as well as the initial PDADMAC, is capable of forming coatings that can be deposited directly on to the hydrophilic and hydrophobic surfaces. At the same time, IPEC demonstrates excellent resistance towards wash-off with water from the both types of surfaces. While bare polycation could be almost eliminated from either the glass or PVC surface, the IPEC with χ = 0.06 retained about 7.5% of its initial deposited mass. This makes such IPECs promising for application as functional cationic coatings. 

## Data Availability

Not applicable.

## Supplementary Materials

The following supporting information can be downloaded at: https://www.mdpi.com/article/10.3390/polym14061247/s1, Figure S1: Structure formulas of polyelectrolytes, Figure S2: Turbidimetric titration curves for the PDADMAC/PSS IPEC mixture with NaCl. C_PDADMAC_ = 4 × 10^−4^ base-mol/L, χ = 0.03 (1); 0.09 (2) and 0.12 (3); pH 7.0, Figure S3: Turbidimetric titration curves for the PDADMAC/PSS IPEC mixture with CaCl_2_. C_PDADMAC_ = 4 × 10^−4^ base-mol/L, χ = 0.03 (1); 0.09 (2) and 0.12 (3); pH 7.0, Figure S4: Dependence of the diffusion coefficient upon the concentration of a solution of PDADMAC (1) and IPEC (2) in a water-salt media. pH 7; C_NaCl_ = 0.05 M, Figure S5: Size distribution densities on the AFM images of the IPEC layers on the glass (a) and PVC (b) substrates.
