# Supplementary material for: Modification of Polydiallyldimethylammonium Chloride with Sodium Polystyrenesulfonate Dramatically Changes the Resistance of Polymer-Based Coatings towards Wash-Off from Both Hydrophilic and Hydrophobic Surfaces
Source: Polymers (Basel). 2022 Mar 19;14(6):1247. doi: 10.3390/polym14061247 (PMC8955630; doi:10.3390/polym14061247)
Supplement: Supplementary file 1 [file polymers-14-01247-s001.zip › polymers-1619807-SI.pdf]

Supplementary Information

For

Modification of polydiallyldimethylammonium chloride with sodium polystyrenesulfonate dramatically change the resistance of polymer-based coatings towards wash-off from both hydrophilic and hydrophobic surfaces

Vladislava A. Pigareva[1], Ivan N. Senchikhin[2], Anastasia V. Bolshakova[1,2] and Andrey V.Sybachin[1,*]

[1] Lomonosov Moscow State University, Chemistry Department, Moscow, 119991, Russia
[2] Frumkin Institute of Physical Chemistry and Electrochemistry, Russian Academy of Sciences, Moscow, 119071, Russia
* Correspondence: sybatchin@mail.ru; Tel.: +74959393114

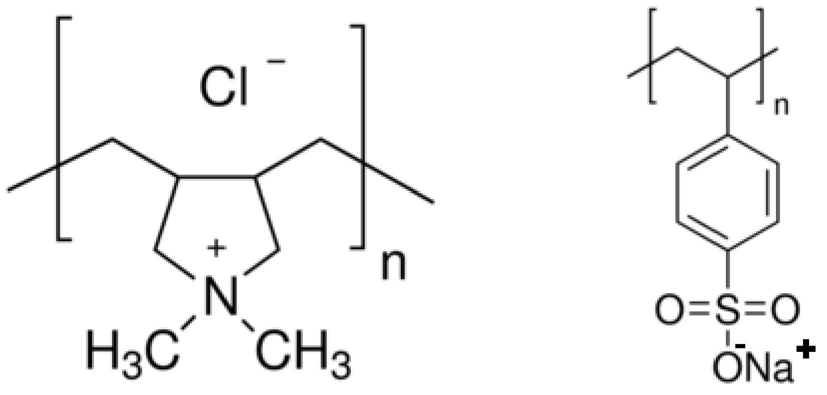

PDADMAC PSS

Figure S1. Structure formulas of polyelectrolytes.

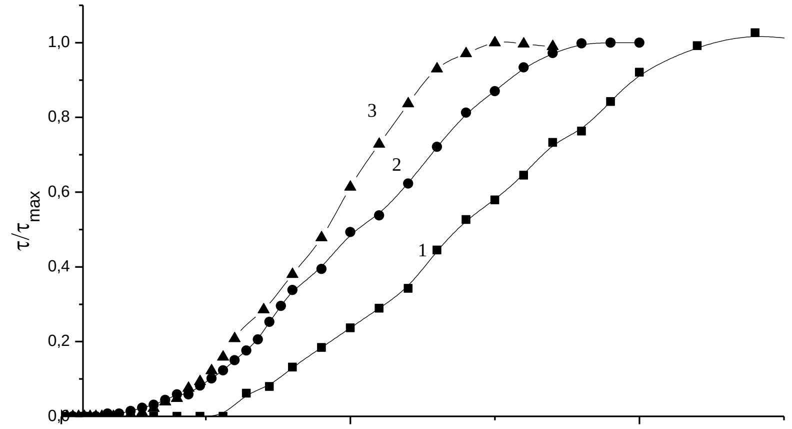

Figure S2. Turbidimetric titration curves for the PDADMAC/PSS IPEC mixture with NaCl. $C_{PDADMAC}$ = 4 × 10^{-4} base-mol/l, χ = 0.03 (1); 0.09 (2) and 0.12 (3); pH 7.0.

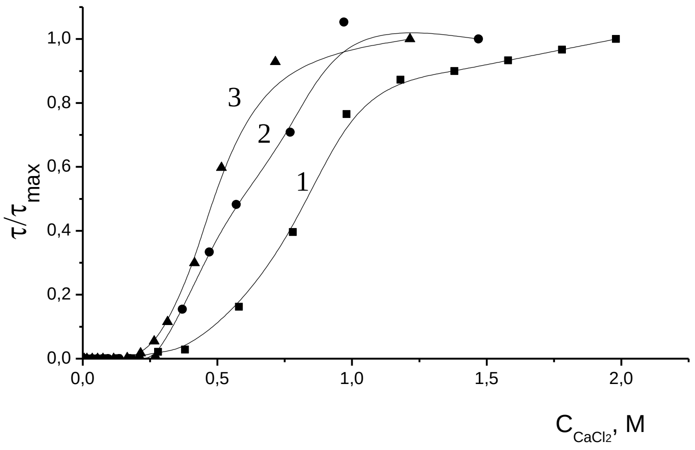

Figure S3. Turbidimetric titration curves for the PDADMAC/PSS IPEC mixture with $CaCl_2$. $C_{PDADMAC} = 4 \times 10^{-4}$ base-mol/l, $\chi = 0.06$ (1); 0.09 (2) and 0.12 (3); pH 7.0.

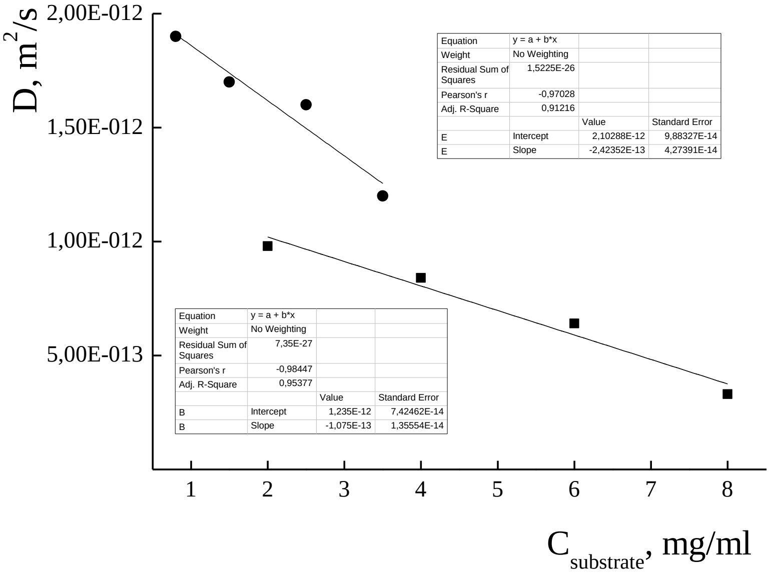

Figure S4. Dependence of the diffusion coefficient upon the concentration of a solution of PDADMAC (1) and IPEC (2) in a water-salt media. pH 7; $C_{NaCl} = 0.05$ M.

Analysis of the average thickness of the IPEC layers on the glass and PVC surfaces was made using Gwyddion software.

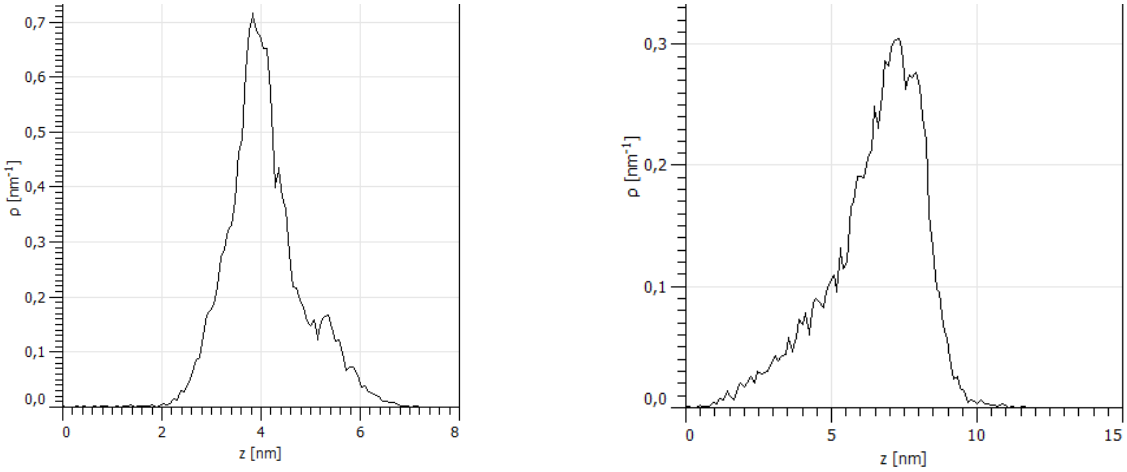

a b

Figure S5. Size distribution densities on the AFM images of the IPEC layers on the glass (a) and PVC (b) substrates
